# Mechanics of epithelial closure over non-adherent environments

**DOI:** 10.1038/ncomms7111

**Published:** 2015-01-22

**Authors:** Sri Ram Krishna Vedula, Grégoire Peyret, Ibrahim Cheddadi, Tianchi Chen, Agustí Brugués, Hiroaki Hirata, Horacio Lopez-Menendez, Yusuke Toyama, Luís Neves de Almeida, Xavier Trepat, Chwee Teck Lim, Benoit Ladoux

**Affiliations:** 1 Mechanobiology Institute, National University of Singapore, Singapore 117411, Singapore; 2 Institut Jacques Monod (IJM), CNRS UMR 7592 and Université Paris Diderot, 75013 Paris, France; 3 Sorbonne Universités, UPMC Univ Paris 06 and CNRS UMR 7598, Laboratoire Jacques-Louis Lions, F-75252 Paris, France; 4 INRIA-Paris-Rocquencourt, MAMBA Team, Domaine de Voluceau, BP105, 78153 Le Chesnay Cedex, France; 5 Institute for Bioengineering of Catalonia, C/ Baldiri Reixac 10-12, 08028 Barcelona, Spain; 6 Department of Biological Sciences, National University of Singapore, 14 Science Drive 4, Singapore 117543 Singapore; 7 Temasek Life Sciences Laboratory, 1 Research Link, National University of Singapore, Singapore 117604, Singapore; 8 Institució Catalana de Recerca i Estudis Avançats (ICREA), Passeig Lluís Companys, 23 08010 Barcelona, Spain; 9 Unitat de Biofísica i Bioenginyeria, Facultat de Medicina, Universitat de Barcelona, and CIBERES, 08036 Barcelona, Spain; 10 Department of Biomedical Engineering, National University of Singapore, 9 Engineering Drive 1, Singapore 117575, Singapore; 11 Department of Mechanical Engineering, National University of Singapore, 9 Engineering Drive 1, Singapore 117575, Singapore

## Abstract

The closure of gaps within epithelia is crucial to maintain its integrity during biological processes such as wound healing and gastrulation. Depending on the distribution of extracellular matrix, gap closure occurs through assembly of multicellular actin-based contractile cables or protrusive activity of border cells into the gap. Here we show that the supracellular actomyosin contractility of cells near the gap edge exerts sufficient tension on the surrounding tissue to promote closure of non-adherent gaps. Using traction force microscopy, we observe that cell-generated forces on the substrate at the gap edge first point away from the centre of the gap and then increase in the radial direction pointing into the gap as closure proceeds. Combining with numerical simulations, we show that the increase in force relies less on localized purse-string contractility and more on large-scale remodelling of the suspended tissue around the gap. Our results provide a framework for understanding the assembly and the mechanics of cellular contractility at the tissue level.

## Abstract (web summary)

Closure of epithelial gaps such as wounds is thought to involve contraction of an actomyosin ‘purse-string’. By creating non-adherent gaps to exclude contributions of adhesive protrusion, the authors find that large-scale tension, more than purse-string contraction, mediates closure.

## Graphical abstract

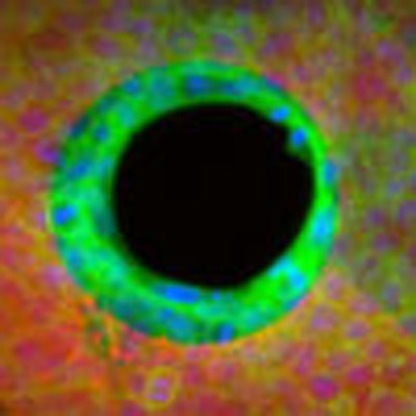

Studying the closure of gaps and discontinuities within multicellular sheets is of great interest because of the important role that it plays in various biological processes such as embryogenesis, tissue morphogenesis and wound healing. Typical examples include dorsal closure in drosophila^1^,^2^, cell extrusion^3^ and wound healing^4^,^5^. When gaps or discontinuities appear in the epithelia, it is widely accepted that there are two major mechanisms that drive the closure of such gaps^6^,^7^,^8^. The first mechanism, termed cell crawling, refers to the protrusive activity of filopodia or lamellipodia at the edge of the gap that propel them into the void^9^,^10^,^11^. The second mechanism, referred to as actin purse-string contraction, is mediated by the coordinated contraction of actin bundles running across multiple cells at the edge of the gap^12^,^13^,^14^.

In many instances, epithelial gap closure occurs over regions where the extracellular matrix (ECM) proteins are either sparsely distributed or even non-existent. In order to close gaps under these environments, epithelial cells can switch from cell crawling mechanism to actin-based purse-string contraction^15^. We have previously reported that keratinocytes migrating on micropatterned lines can form suspended epithelial bridges that rely on contractile actin bundles over regions devoid of ECM proteins to close gaps and maintain epithelial integrity^16^. However, as both cell crawling and actin purse-string mechanisms co-exist during gap closure, this can induce discontinuities in the actin organization around the gap because of the presence of protrusive extensions as well as contractile actin bundles^7^,^10^,^17^,^18^. Both mechanisms could thus influence each other during epithelial resealing. In addition to lamellipodia extensions towards the gap, the assembly of discontinuous supracellular contractile actin cables connected to the substrate through focal adhesions promotes efficient wound closure by compressing the underlying substrate^19^. Because such mechanisms rely on cell–substrate interactions, it is difficult to understand how gaps close in situations where the ECM is heterogeneous and/or poorly adherent. In such cases, the purse-string contraction of actin cables appears to be the crucial mechanism for gap closure but it remains poorly characterized.

Here, by using micropatterned substrates^20^, we study the closure of circular gaps devoid of ECM protein and functionalized with a cell non-adhesive polymer within sheets of keratinocytes. We find that closure of such non-adherent gaps is driven exclusively by contraction of multicellular actin-based cables. The ability to close these gaps is determined by geometrical cues such as size and curvature of the gap as well as intact intercellular junctions. Traction force microscopy (TFM) and numerical simulations suggest strong reinforcement of the contractile force driving the gap closure. Such reinforcement appears to be originating from large-scale remodelling of cells at the gap edge.

## Results

### Closure of non-adhesive gaps by purse-string contraction

**Figure 1:**
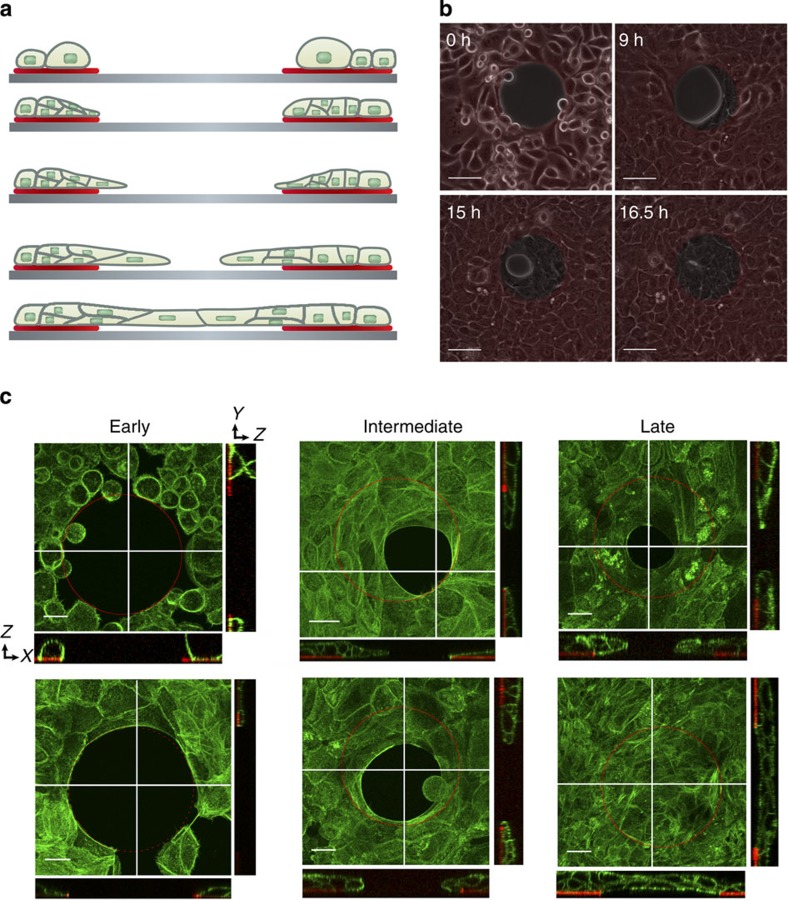
HaCaT cells close a 100 μm diameter non-adhesive gap using actin purse-string contraction. (**a**) Schematic and (**b**) phase contrast images showing closure of a circular non-adhesive gap with time. Cells are initially rounded and confined to the μcontact printed adhesive fibronectin (red) region but with time gradually spread and close the non-adhesive circular gap. (**c**) *z*-projection of confocal images and the corresponding *xz* and *yz* sections (along the white lines) of cells stained for F-actin during ‘early’, ‘intermediate’ and ‘late’ stages of closure of the circular non-adhesive gap (red circle). The top panels represent the approximate start of the phase and bottom panels mark the approximate end of each phase. Scale bars, (**b**) 50 μm and (**c**) 20 μm.

Based on our previous experiments, we hypothesized that circular non-adhesive gaps within keratinocyte cell sheets would promote the formation of contractile purse-strings composed of actin filaments and thus help us to elucidate the mechanics of multicellular actin-based purse-string contraction within a well-defined environment. To test this hypothesis, HaCaT cells were seeded on micropatterns consisting of a 100 μm diameter circular non-adhesive gap (rendered non-adhesive with Pluronics, see methods) at the centre of a large (~800 μm) fibronectin-coated square. Surprisingly, we observed collective cellular movements towards the centre of the gap solely driven by actomyosin contraction ending in complete gap closure. Initially, cells confined themselves to the adhesive region leaving the non-adhesive gap empty (Fig. 1a,b and Supplementary Movie 1). With progression of time, they gradually moved in and closed the gap. Although no lamellipodia were observed at the advancing cell front, the edge showed a strong contrast in phase images suggesting that the closure was driven by contraction of actin cables (Fig. 1b). Staining for filamentous actin at different time points not only confirmed strong accumulation of F-actin at the edge but also allowed us to broadly divide the closure event into different stages (Fig. 1c). About 1–2 h after initial seeding (Fig. 1c, early stage, left panel), cells adhered to the fibronectin-coated region of the substrate. Gradually, cells started spreading and formed filopodia and lamellipodia over the fibronectin-coated pattern. After complete spreading, cells at the gap edge displayed strong accumulation of F-actin resulting in the formation of discontinuous actin cables around the gap. This was followed by an anisotropic closure of the gap with the formation of multicellular actin cables that partly extend over the non-adhesive region on one side and multiple contractile cables still connected to the substrate on another side (Fig. 1c, intermediate stage, middle panel). With further progression of time, the actin cables contracted rapidly resulting in complete closure and formation of a suspended epithelial sheet over the non-adhesive gap (Fig. 1c, late stage, right panel).

**Figure 2:**
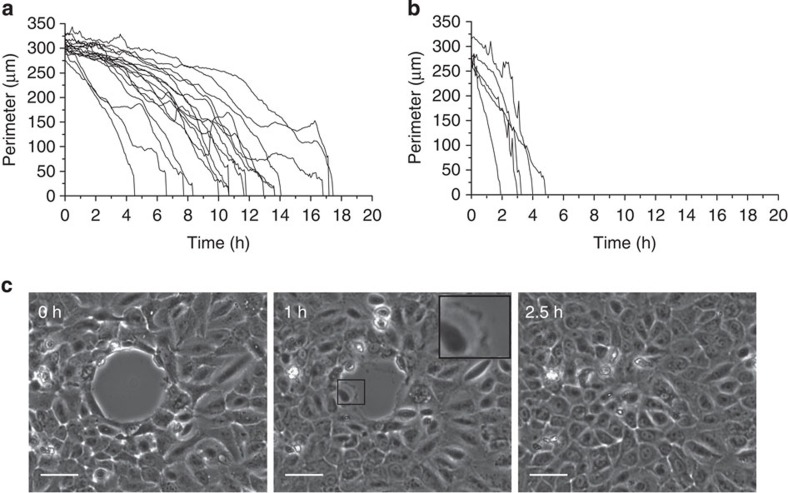
Closure dynamics of adhesive and non-adhesive gaps. Closure of (**a**) non-adhesive gaps (mediated by actin cable contraction) and (**b**) adhesive gaps (mediated by cell crawling mechanism). (**c**) Phase contrast images of HaCaT cells closing an adhesive epithelial gap created after the removal of a micropillar stencil. Inset shows cells forming lamellipodia. Scale bars, (**c**) 50 μm.

To analyse whether these different stages reflected in the closure dynamics of the non-adhesive gap, the perimeter of the gap was tracked over time (Fig. 2a). The plot not only showed a large variation in the total time taken for closure from one gap to another (~4.5 to 17.5 h) but also suggested that the closure was highly non-linear and occasionally fluctuated. Indeed, although the average time taken for complete closure of the gap was 11.7±3.7 h (*n*=17, mean±s.d.), the average time required for closure of the initial approximately one-third of the gap was 6.77±2.97 h, whereas the latter approximately two-third of the gap took 4.9±1.67 h to close. This strongly suggests that the slow first phase represents not only the time taken for the cells to spread but also the time required for cellular re-organization around the gap to build-up multiple contractile cables. In contrast, the rapid second phase represents a more coordinated and efficient contraction of the suspended cell front. The fluctuations in the perimeter suggest that the purse-string does not contract continuously but rather relaxes intermittently. As a control, we then compared this with closure of a 100 μm diameter adhesive gap induced by the removal of a micropillar stencil^10^ (Fig. 2b). The gap closure was completed in 3.6±1.2 h (*n*=16), which is comparable to the speed of a migrating HaCaT monolayer^16^. The closure process was mainly driven by cell crawling with lamellipodia extension with some reinforcement of actin at the edge of the gap (Fig. 2c and Supplementary Movie 2). This result shows that our experimental system is well-designed to exclusively study the influence of actomyosin purse-string contraction on epithelial gap closure.

### Curvature regulates purse-string contraction

**Figure 3:**
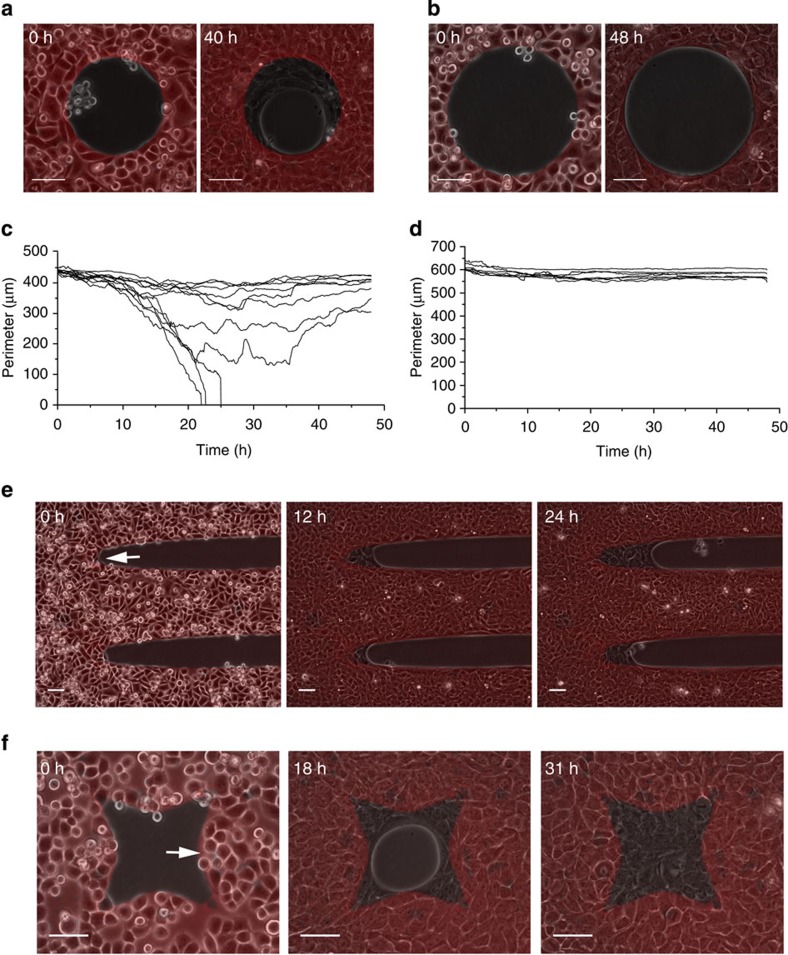
Closure of non-adhesive gaps is determined by the size and curvature of the gap. (**a**,**b**) Phase contrast images and (**c**,**d**) closure dynamics of 150 and 200 μm diameter non-adhesive gaps, respectively. (**e**) Elliptical non-adhesive gaps (minor/major axis ratio=100:1,000 μm) are partially closed in regions with high curvature (arrow) and (**f**) closure of non-adhesive gaps with negative curvature (arrow) is delayed. Scale bars, 50 μm.

To further investigate the effect of gap curvature on purse-string contraction, we varied the dimensions and the shapes of the non-adhesive regions. First, cells were seeded on non-adhesive gaps with diameters of 150 and 200 μm. In contrast to the 100 μm non-adhesive gaps that cells were always able to close, the 150 μm diameter non-adhesive gaps could be closed only in ~20% of cases. However, in most instances (~80% of the cases), the cell front advanced to close the gap only partially (Fig. 3a and Supplementary Movie 3). On the 200 μm diameter gaps, the cell front barely advanced into the gap and no closure was observed (Fig. 3b and Supplementary Movie 4). These observations were further supported by the plot of the change in perimeter of the gap with time (Fig. 3c,d). This relationship between epithelial closure and gap curvature not only emphasizes the role of actin-based contractile forces at the edge of the gap in driving the closure process but also highlights the existence of a critical diameter of ~150 μm, above which such contraction is ineffective. Also, as a larger gap can be interpreted as a geometrical feature with lower curvature, we hypothesized that the curvature of the ECM geometry influences the stability of actin cable formation as well as its efficient contraction. To test this hypothesis, we seeded cells on two different patterns. The first was an elliptical non-adhesive gap whose minor and major axes were ~100 and ~1,000 μm, respectively (Fig. 3e). As in the previous instances, cells were initially confined to the adhesive fibronectin region leaving the elliptical non-adhesive region empty. With time, cells started migrating into the gap from one end of the ellipse that displayed very high curvature (arrow, Fig. 3e and Supplementary Movie 5). Interestingly, the cell front reached an ‘equilibrium-like’ state and stopped migrating after ~24 h. The second pattern consisted of a non-adhesive gap with negative curvatures (convex projections into the gap, arrow in Fig. 3f) to the cell sheet. On this pattern, cell sheets initially migrated to close the corners of the gap that provided high curvature (Supplementary Movie 6). However, movement of the cell front was delayed across the sides of the gap with a strong negative curvature because of their convexity. Eventually, a circular actin-based purse-string was formed that resulted in gap closure. Together, these results suggest that geometrical cues such as curvature can strongly influence the formation, stability and contraction of actin cables.

### α-Catenin is essential for closure of non-adhesive gaps

**Figure 4:**
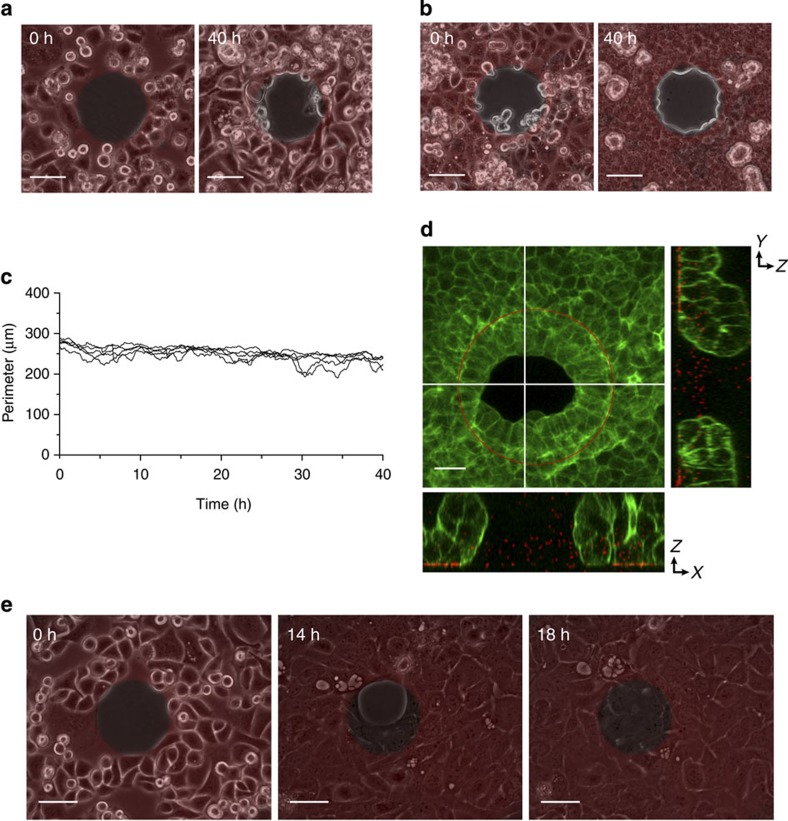
Strength and integrity of intercellular adhesion regulate closure of non-adhesive gaps. (**a**) Phase contrast images of α-catenin knockdown HaCaT cells on 100 μm diameter non-adhesive gaps. (**b**) Phase contrast images, (**c**) closure dynamics and (**d**) confocal images of actin staining of MDCK cells on 100 μm diameter non-adhesive gaps. (**e**) HaCaT cells close 100 μm non-adhesive gaps in the presence of 2 mM thymidine (proliferation inhibitor). Scale bars, (**a**,**b**,**e**) 50 μm and (**d**) 20 μm.

Epithelial reshaping processes involve a remodelling of adherens junctions^21^,^22^,^23^,^24^. We hypothesized that efficient force transmission through supracellular contractile actin cables would require intact intercellular adhesion during gap closure as described by previous studies^14^,^25^. To test this, we generated HaCaT cells that were stably knocked down for α-catenin (α-HaCaT) and desmoplakin (dsp-HaCaT), which are considered as key adaptor molecules at the adherens junctions and desmosomes, respectively^26^,^27^,^28^. α-HaCaT cells did not form intercellular junctions and failed to close the non-adhesive gaps (Fig. 4a and Supplementary Movie 7). Instead, these cells migrated individually and single α-HaCaT cells occasionally stretched across the gap. In contrast, dsp-HaCaT cells could efficiently close the non-adhesive gap (Supplementary Movie 8) in a manner that was indistinguishable from wild-type HaCaT as well as HaCaT cells expressing non-targeting small hairpin RNA (shRNA) (Supplementary Movie 9). This suggested that α-catenin but not desmoplakin was indispensable for coordinated contraction of the purse-string. This is consistent with recent studies that emphasize the role of adherens junctions in establishing tissue level tension^29^. The importance of intercellular adhesion was also evident from the fact that simple epithelial cells such as MDCK that demonstrate a more ‘viscous-like’ behaviour because of higher cell–cell rearrangement (compared with HaCaT)^16^ could not close a 100 μm non-adhesive gap even after ~40 h despite reaching very high densities and eventually over confluence (Fig. 4b,c and Supplementary Movie 10). In fact, the partial closure of the gap that did occur was a result of cell overgrowth resulting in formation of three-dimensional-like tissue structures (Fig. 4d) as previously reported^30^. Consequently, even though these findings demonstrate the importance of tensile stress through intercellular adhesions during epithelial gap closure, we could not exclude a possible role of proliferative pressure from the surrounding tissue. To test this, we inhibited cell proliferation in this process. When cell proliferation was inhibited, the 100 μm diameter non-adhesive gap could still be closed, although the closure was slightly delayed (Fig. 4e and Supplementary Movie 11). This delay was not surprising as inhibition of proliferation would also increase the time taken for cells to completely occupy the adhesive region before migrating into the non-adhesive region. Thus, cell proliferation does not appear to be the major driving force in our model of epithelial gap closure.

### TFM of contracting purse-string

**Figure 5:**
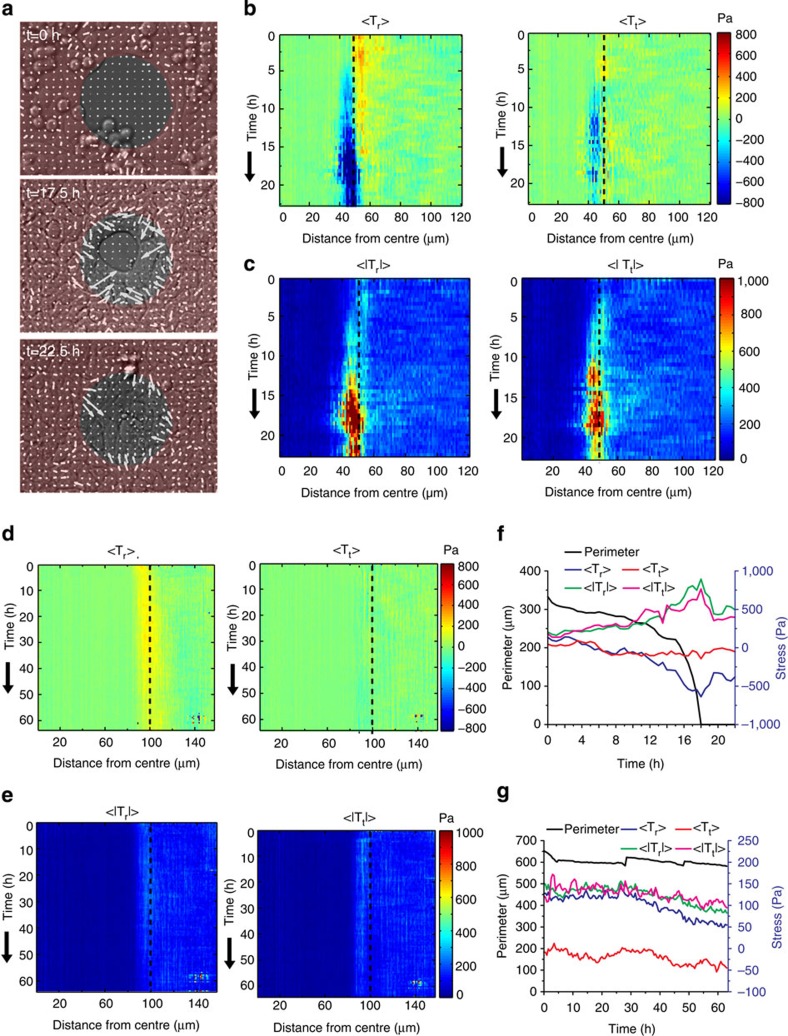
Traction force microscopy of HaCaT cells on 100 and 200 μm diameter non-adhesive gaps. (**a**) Evolution of traction stresses with time as the non-adhesive gap is closed. Arrows depict both the direction and magnitude of the stresses exerted by cells on the substrate. Kymographs showing (**b**,**d**) vectorial and (**c**,**e**) scalar average of radial (T_r_, left panel) and tangential (T_t_, right panel) stresses as a function of distance from the centre of 100 and 200 μm gaps, respectively. The dashed dotted line represents the gap edge. Average stresses along a 20 μm wide strip centred on the edge of the (**f**) 100 and (**g**) 200 μm diameter non-adhesive gap.

**Figure 6:**
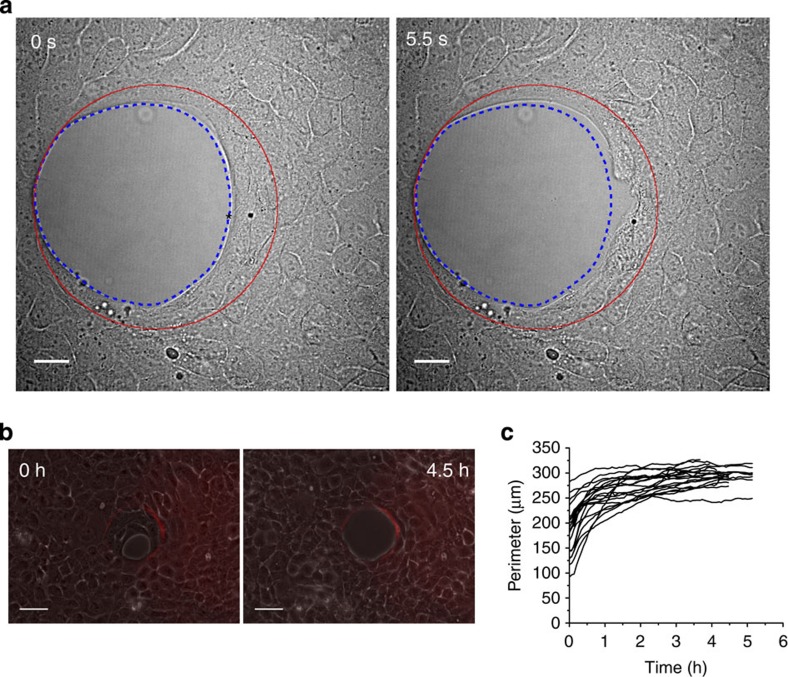
Closure of non-adhesive gap requires actomyosin contractility. (**a**) Laser ablation of the actin cables at the edge results in immediate relaxation of the cell front. The asterisk represents the point of laser ablation, red circle represents the edge of the 150 μm diameter non-adhesive gap and blue dotted line is the edge of the cell front before laser ablation. (**b**) Phase contrast images and (**c**) relaxation dynamics of a partially closed 100 μm diameter non-adhesive gap after addition of 50 μM blebbistatin. Scale bars, (**a**) 20 μm and (**b**) 50 μm.

We thus considered that the closure of these epithelial gaps could result from a ‘tug of war’ between the contracting actin-based purse-string at the edge of the gaps and the resistive forces offered by the cells adherent to the fibronectin-coated substrate. In such a model, the forces generated by the contracting actin-based purse-string should reflect in the traction forces exerted by cells in the vicinity of the edge of the non-adhesive gap. To test this hypothesis, we used TFM. Non-adhesive gaps of diameters 100 and 200 μm were patterned on soft silicone elastomeric substrates functionalized with fluorescent beads as described previously^16^,^31^. Closure of the gap and deflection of beads (Fig. 5a) were tracked over time and unconstrained traction stresses were calculated from the bead displacement as previously described^32^. Radial stresses pointing inward and tangential stresses directed counter-clockwise were considered negative. Kymographs of vectorial and scalar average of radial (<T_r_>, <|T_r_|>) and tangential (<T_t_>, <|T_t_|>) stresses as a function of distance from centre of the gap showed that the maximal stresses were localized to ~10 μm on either side of the edge of the gap (Fig. 5b–e). Accordingly, average radial and tangential stresses in this ‘strip’ or region of interest were plotted as a function of time for further analysis. For the 100 μm diameter gap, <T_r_> was initially positive (directed outwards) consistent with the idea of cells that were spreading or crawling to reach the edge of the gap (Fig. 5f). With the formation of a contractile purse-string and movement of cells into the gap, <T_r_> became increasingly negative (pointing inwards) and reached a nadir (approximately −600 Pa) just about the time the gap closed (Fig. 5f). After the closure of the gap, <T_r_> showed a slight increase but remained negative suggesting that cells bridging the gap exhibited residual tension despite the dissolution of the actin-based purse-string. The average tangential stress <T_t_>, on the other hand, fluctuated but remained close to zero throughout the observation period (Fig. 5f). However, the scalar average of the radial (<|T_r_|>) and tangential (<|T_t_|>) stresses increased and reached a peak just around the time the gap closed after which they showed a sudden fall. The force increase in the radial direction suggests a reinforcement of the contracting actin cable as gap closure progresses. Further support for such a force balance between cell–substrate adhesion and contracting actin cables came from estimation of the critical length scale based on estimated cell adhesion energy density *γ* (J m^−2^). The total work done to move a ‘suspended’ cell sheet of radius *R* over the non-adhesive region by a further distance *dr* is given by *γ*(2*πR*)*dr.* The radial force within the actin cable should be large enough to overcome this energy barrier, that is, *Fdr*=*γ*(2*πR*)*dr*. The maximal radial force within the cable estimated by TFM is ~4.0 μN (~650 Pa over ~6,300 μm^2^ area of the strip of interest). Although data for *γ* for keratinocytes are not available in the literature, the estimated value for fibroblasts on fibronectin is ~15 mJ m^−2^ (ref. 33). This gives a critical length scale *R* equal to ~40 μm for closure that correlates well with our experimental observations. For the 200 μm diameter gap, minimal stresses were detected at the edge of the hole that corroborated with the fact that the cell front had barely migrated into the gap (Fig. 5g). To directly test the influence of such contractile tension on gap closure, we laser ablated actin bundles at the edge (Fig. 6a and Supplementary Movie 12) and inhibited actomyosin contractility with blebbistatin (Fig. 6b,c and Supplementary Movie 13). Both experiments induced a relaxation of a partially closed non-adhesive gap and thus supported the idea of force transmission from the contracting actin cable to cells adherent to fibronectin at the edge of the gap.

### Modelling the closure of a non-adhesive circular gap

**Figure 7:**
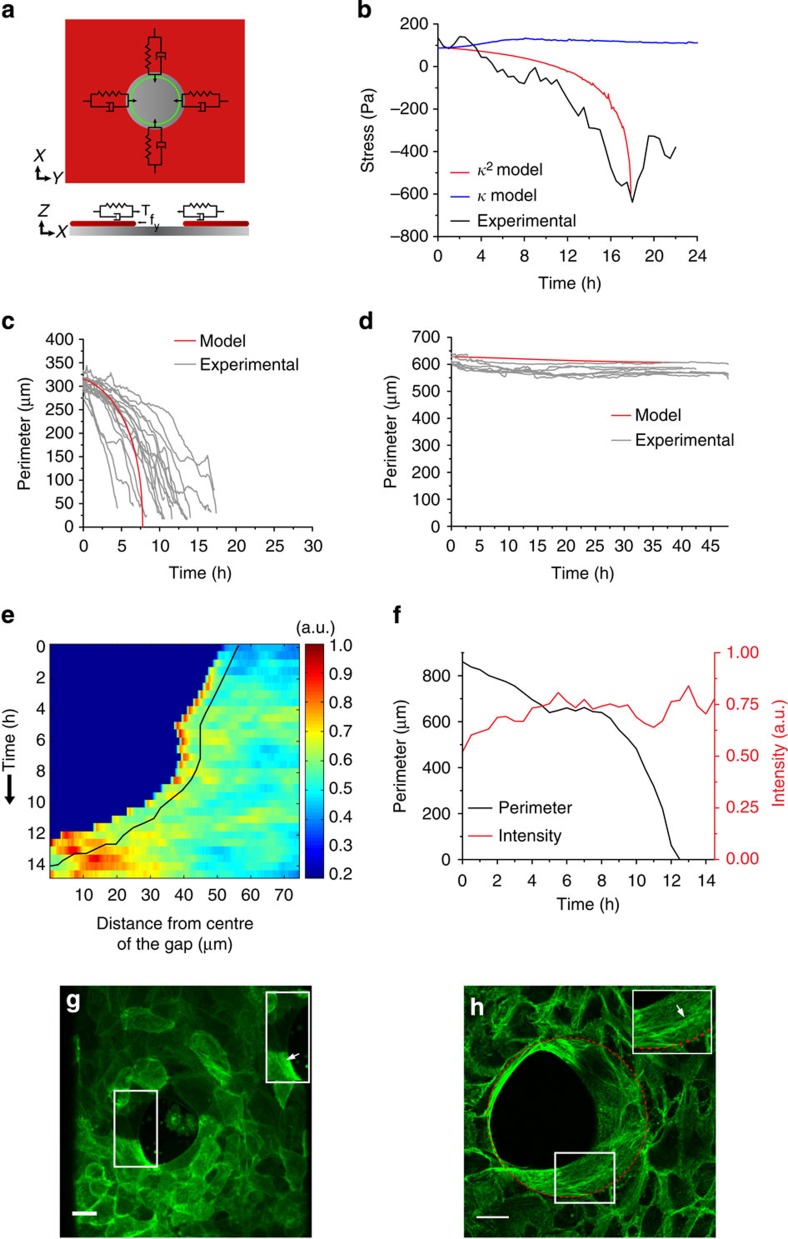
Numerical simulation of the gap closure. (**a**) Schematic of the model showing a balance between the tension in the actin cable (green) *T* and the frictional force *f*_y_ arising from tissue adhesion to substrate on the fibronectin (red) and the tissue itself represented as a viscoelastic material. (**b**) Average radial stress at the edge of the gap predicted by the model with (*κ*^2^ model) or without (*κ* model) reinforcement of tension in the actin purse-string. Change in the perimeter of the gap over time predicted by the tension reinforcement model for (**c**) 100 μm and (**d**) 200 μm diameter gaps. Simulation values have been scaled to fit the experimental data. (**e**) Kymograph of actin intensity from the centre of the gap. Black line is a guide to show an ~12 μm wide strip along the edge of the cell front. (**f**) Change in average intensity of actin with time in the strip of interest bounded by the black line in **e**. (**g**) Actin network visualized using GFP-lifeact-expressing HaCaT cells and (**h**) basal confocal section of cells fixed and stained for actin showing strong re-organization within cells localized to the edge of the gap (insets). Scale bars 20 μm.

To further test our hypothesis of a force balance between contractile forces of the actin cable and resistive viscoelastic responses of the remaining adherent cells, we developed a mechanical model that captures our experimental findings. The tissue was modelled as a solid viscoelastic Kelvin-Voigt material, and the interaction with the adhesive part of the substrate was modelled so that the velocity of the tissue is zero if the force exerted on the tissue is less than a threshold *f_y_*, and linearly increases with the force above this threshold (Fig. 7a, Supplementary Note 1 and Supplementary Fig. 1). Assuming that the actomyosin structure on the border of the gap acts as a cable under tension *T*, the resulting force per unit length exerted on the tissue is *Tκ*, where κ is the local curvature and is the normal vector to the border directed towards the interior of the gap^34^,^35^. Numerical simulations showed that a constant tension (κ model) could not account for the increasing stress at the edge of the gap; however, a simple model of cable reinforcement (tension increasing linearly with curvature, κ^2^ model) could predict this experimental observation (Fig. 7b). Such a tension reinforcement model (κ^2^ model) together with a threshold friction force could not only account for the fact that 100 μm but not 200 μm diameter gaps close but could also explain the experimentally observed increase in closure velocity for the 100 μm diameter gaps with time (Fig. 7c,d). To understand the origin of the reinforcement, we performed live-cell imaging of HaCaT cells stably expressing GFP-lifeact (Supplementary Movie 14). Although there was some increase in the intensity of actin at the edge of the suspended cell front (Fig. 7e,f), a more prominent observation was the large-scale re-organization of the actin cytoskeleton within the cells around the gap directed tangentially to the gap (Fig. 7g, inset). Such a re-organization was also consistently observed in cells fixed and stained for actin immediately before gap closure (Fig. 4h, inset). We believe that this re-organization of actin cytoskeleton at tissue level probably plays a significant role in reinforcing overall contractility of the suspended cell front.

## Discussion

In view of this relationship, our assay determines that fine tuning between cell–substrate interactions and strengthening of multicellular actin cables through intercellular adhesions remains crucial to understand epithelial gap closure. Indeed, the mechanistic characterization of actin-based purse-string-mediated closure of epithelial gaps has remained elusive because of the lack of an assay that could faithfully and consistently reproduce it *in vitro*. Most of our understanding about this mechanism of gap closure comes from experiments involving laser ablation of cell clusters *in vitro*^13^,^19^ or *in vivo*^34^,^36^. However, in both experimental systems it is almost impossible to segregate the individual contributions of cell crawling and purse-string contraction mechanisms to the overall closure process. Furthermore, in the absence of a direct ‘readout’, quantification of forces generated by the actin cable is indirectly inferred from the retraction velocity of tissues following laser ablation. In contrast, our experimental setup provides a novel platform to isolate and characterize the mechanics of purse-string contraction during epithelial gap closure.

Using a similar experimental approach, Kim *et al*. recently studied traction forces in cell monolayers that surrounded but never invaded non-adherent islands^37^. They found that cells enveloping the islands exerted radial forces pointing uniformly away from the island. This intriguing behaviour, which they called kenotaxis, was independent of the orientation of the cell velocity vector and principal stress directions. Using cells that are able to invade non-adherent islands, here we found traction force patterns that are similar in orientation but opposite in sign. Indeed, we found that forces are mostly radial but point towards the island rather than away from it (note that Kim *et al*. reported forces exerted by the gel on the cells rather than by the cells on the gel as we did here). These findings show that fundamentally distinct mechanical scenarios are at play in cells that invade gaps compared with cells that only envelop them. This is also reflected by the fact that, unlike cells enveloping non-adherent islands, cells that invade the islands show non-negligible tangential tractions (Fig. 5b,c)^19^.

Although closure mediated by contraction of actin cables appears to be slower and inefficient compared with cell crawling, it is probably important in situations where cell–ECM contact is either poor or non-existent. Indeed, purse-string contraction appears to be the dominant mechanism of wound/gap closure in embryos, which exhibit low levels of ECM providing little cell–substrate contact for migration^14^. Furthermore, efficient formation and contraction of such actin-based purse-strings appears to be limited to cell types that are involved in wound healing (for example, skin keratinocytes and embryonic epidermal cells) as simple epithelial cells such as MDCK are unable to close similar non-adhesive gaps efficiently. Such specificity could arise from differences in the organization and strength of intercellular adhesion in these cell types. Our findings demonstrate the importance of contractile mechanical forces generated by large-scale re-organization of actin cytoskeleton in tissue remodelling during epithelial closure and thus provide new mechanistic insights into these processes.

## Methods

### Cell culture and reagents

HaCaT cells (Cell Lines Service) and MDCK cells were maintained in DMEM supplemented with 10% fetal bovine serum and antibiotics. Protein expression in HaCaT cells was depleted by retrovirus-mediated introduction of shRNA into cells, as described previously^16^. The target sequences used were 5′- GACTTAGGAATCCAGTATA -3′ (for α-catenin) and 5′- GTGACCAACTTGTCCTCAA -3′ (for desmoplakin). As a control, shRNA with the non-targeting sequence (5′- ATAGTCACAGACATTAGGT -3′) was used. For inhibition of proliferation, HaCaT cells were first maintained in 2 mM thymidine for 24 h following which cells were trypsinized, seeded on the patterns and imaged in medium containing 2 mM thymidine.

### Microcontact printing and microscopy

Patterned silicon wafers for soft lithography were prepared using SU-8 photoresist^20^,^38^. For imaging the closure dynamics of the gaps, patterns were microcontact printed on non-culture treated Petri dishes (Greiner) and blocked with 0.2% Pluronics. Cells were seeded at high density and allowed to adhere for 1–2 h to completely fill the adhesive region of the pattern. Floating cells were washed off and the closure was imaged on a Biostation (Nikon). For closure of circular adhesive epithelial gaps by cell crawling, micropillar stencils removal assay was performed^10^. The closure dynamics (contour of the non-adhesive gap) was obtained using the ABsnake plugin for Image J, fitted with an ellipse using MATLAB. For confocal imaging, patterning was done on glass bottom Petri dishes spin coated with a thin layer of polydimethylsiloxane (PDMS). Cells were imaged using a laser confocal microscope (Leica). Images were processed in Image J to enhance contrast. Laser ablation was done using ultraviolet laser (355 nm, Minilite II, Continuum) mounted on a confocal microscope (Nikon A1R) using a × 40 water immersion objective^16^.

### Traction force microscopy

Substrates for TFM were prepared from soft silicone gels^16^,^39^. Briefly, soft silicone gels were mixed in a ratio of 1:1 and spin coated on a glass bottom Petri dish and cured. The gels were silanized by incubating in 5% (3-aminopropyl)triethoxysilane (APTES, Sigma) in ethanol for 5 min. The substrates were dried and incubated with 100 nm carboxylated fluorescent beads (Invitrogen) for 5 min, washed and dried. The beads were passivated with 100 mM Tris base (1st Base) in water and dried. Fibronectin pattern was stamped on the soft silicone gels using a water-soluble polyvinyl alcohol (PVA) membrane as an intermediate substrate^31^. The gels were finally blocked with 0.2% Pluronics solution. Cells were seeded on the patterns and imaged on an Olympus inverted microscope. After imaging, cells were lysed and the ‘stress free’ image of the beads was obtained. The unconstrained stresses were computed using custom written MATLAB codes^32^.

## Additional information

**How to cite this article**: Vedula, S. R. K. *et al*. Mechanics of epithelial closure over non-adherent environments. *Nat. Commun.* 6:6111 doi: 10.1038/ncomms7111 (2015).

## Supplementary Material

Supplementary Figure, Supplementary Note and Supplementary References Supplementary Figure 1, Supplementary Note 1 and Supplementary References

Supplementary Movie 1 HaCaT cells closing a 100 μm diameter non-adhesive gap. Scale bar 50 μm.

Supplementary Movie 2 HaCaT cells migrating into a 100 μm diameter adhesive gap after removal of the micropillar stencil. Scale bar 50 μm.

Supplementary Movie 3 HaCaT cells partially closing a 150 μm diameter non-adhesive gap. Scale bar 50 μm.

Supplementary Movie 4 HaCaT cells unable to close a 200 μm diameter non-adhesive gap. Scale bar 50 μm.

Supplementary Movie 5 HaCaT cells partially closing elliptical non-adhesive gaps. Scale bar 50 μm.

Supplementary Movie 6 Non-adhesive gaps with 'positive curvature' (convex) delay closure. Scale bar 50 μm.

Supplementary Movie 7 a-catenin knockdown HaCaT cells are unable to close a 100 μm diameter non-adhesive gap. Scale bar 50 μm.

Supplementary Movie 8 Desmoplakin knockdown HaCaT cells are able to close 100 μm diameter non-adhesive gap. Scale bar 50 μm.

Supplementary Movie 9 Control HaCaT cells expressing non-targeting shRNA are able to close a 100 μm diameter non-adhesive gap. Scale bar 50 μm.

Supplementary Movie 10 MDCK cells are unable to close a 100 μm diameter non-adhesive gap. Scale bar 50 μm.

Supplementary Movie 11 HaCaT cells close 100 μm diameter non-adhesive gaps in the presence of 2mM thymidine that inhibits cell

Supplementary Movie 12 Addition of blebbistatin to partially closed100 μm diameter non-adhesive gap results in 'opening up' of the gap. Scale bar 50 μm.

Supplementary Movie 13 Laser ablation of the cell front in a partially closed non-adhesive gap results in rapid relaxation of the cell edge. Scale bar 20 μm.

Supplementary Movie 14 Z-projection of live cell imaging of HaCaT cells expressing lifeact-GFP using spinning disc confocal microscopy. Scale bar 20 μm.
