# Supplementary material for: Mechanics of epithelial closure over non-adherent environments
Source: Nat Commun. 2015 Jan 22;6:6111. doi: 10.1038/ncomms7111 (PMC4309897; doi:10.1038/ncomms7111)
Supplement: Supplementary Figure, Supplementary Note and Supplementary References — Supplementary Figure 1, Supplementary Note 1 and Supplementary References [file ncomms7111-s1.pdf]

Supplementary Information

Supplementary Figure 1

Numerical Simulation Model

- **Geometry:**

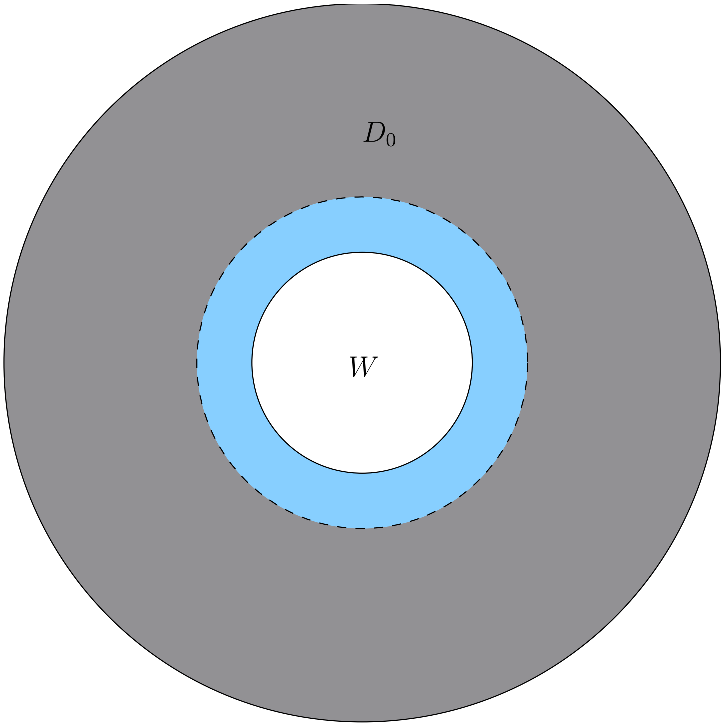

Supplementary Figure 1 Gap closure in a circular domain Ω with initial tissue position D_0 (in gray); D_0 coincides with the localization of the adherent substrate. The actual tissue position is $D = \Omega \backslash W$.

Supplementary Note 1

- **Cell/cell interaction: visco-elastic solid**
  The tissue is modeled as a Kelvin-Voigt visco-elastic solid material: its stress tensor σ is given by:

$$\sigma = 2\eta D(v) + 2G\varepsilon - pI,$$

where η is the viscosity and G is the Young modulus; $D(v) = 1/2(\nabla v + \nabla v^T)$ is the symetrized part of the velocity gradient, ε is the deformation tensor, p is a pressure, and I

is the identity matrix. Cells are considered as a tridimensional incompressible material ; however, in the bidimensional configuration studied here, where the height of cells is negligible compared to the size of the monolayer, cells can adjust their height by spreading or contracting: as a 2D material, the tissue is compressible, any vertical deformation is compensated by the pressure term. The 2D constitutive equation of the monolayer can be written with effective viscosity and Young modulus (still denoted as η and G for the sake of simplicity):

$$\sigma = 2\eta D(v) + 2G\varepsilon \text{ on } D.$$

- **Cell/substrate interaction: adhesive forces**
  Cell/substrate interactions forces are modeled as external forces F_S for the tissue, so that the momentum balance expresses the balance between cell/cell and cell/substrate interactions:

$$-\nabla \cdot \sigma = F_S \times \chi_{D_0} \text{ on } D,$$

where χ_{D_0} is the indicator function of the adhesive substrate D_0. Cells can move on D_0 either because they are pulled by other cells ($-\nabla \cdot \sigma$ term in the momentum balance) or because of the forces acting on the boundary of the tissue (described below). We introduce a threshold force f_Y such that cells adhere to the substrate with zero velocity when they are pulled with a force that is less than this threshold, and slip with a friction force proportional to velocity otherwise. By analogy with visco-plastic models[1], this behavior is modeled as :

$$-\nabla \cdot \sigma = \left(-\frac{f_y v}{\|v\|} - C_F v\right)\chi_{D_0} \text{ if } v \neq 0,$$

$$\|\nabla \cdot \sigma\| \leq f_y \text{ otherwise.}$$

This expression is regularized for easier numerical resolution, in the same spirit as in[2]:

$$-\nabla \cdot \sigma = \left(-f_y \frac{1 - \exp(-m\|v\|)}{\|v\|} v - C_F v\right)\chi_{D_0}.$$

As the numerical parameter m increases, the regularized force converges towards the exact one.

- **Forces on the moving boundary**: The closing mechanisms acting on the border ∂W of the tissue are modeled as a boundary condition for the stress tensor:

$$\sigma \cdot n = (f_L + g(\kappa))n, \text{ on } \partial W.$$

where n is the normal vector on the boundary, directed toward the exterior of the tissue, f_L is the lineic density of forces exerted by the lamellipodia and $g(\kappa)$ is a function depending on the local curvature κ, that represents the tension exerted by the acto-myosin structure. In this work, we will assume that $g(\kappa)$ is a power law given by:

$$g(\kappa) = -\gamma\ sign(\kappa)\ |\ \kappa\ |^{\alpha}$$

where γ and α are positive real parameters describing the tension. The power law is written in this complex way since α is real and κ may be negative (with the convention we adopted, it is negative in case of a circular gap). We remark that this work concerns the case $f_L = 0$ since the interior of the gap is non-adherent, (no traction force due to lamellipodia in that area).

- **Closure of the model:** Eulerian formalism. The evolution of the deformation tensor is equal to the strain rate tensor:

$$\frac{\mathcal{D}\varepsilon}{\mathcal{D}t} = D(v),$$

where $\frac{\mathcal{D}\varepsilon}{\mathcal{D}t} = \partial_t\varepsilon + (v \cdot \nabla)\varepsilon + \varepsilon W(v) - W(v)\varepsilon - a(D(v)\varepsilon + \varepsilon D(v))$ is an objective derivative, and $W(v) = 1/2(\nabla v - \nabla v^T)$. This formalism is equivalent to the classical lagrangian formulation in linear elasticity and has the advantage of being compatible with a numerical method designed for visco-elastic fluids previously developped in the team.

- **Numerical resolution:** We take advantage of the circular geometry to solve the equations in polar coordinates, with only one space dependance along the radial coordinate r ; the unknowns of the problem are the velocity $v(t,r)$ and elastic deformation $\varepsilon(t,r)$. Based on a time discretization with time step Δt, the time derivatives are approximated with an Euler scheme:

$$\partial_t\varepsilon \approx \frac{\varepsilon^{n+1} - \varepsilon^n}{\Delta t}$$

where n and $n+1$ denote two successive time steps. From an initial condition at time $t = 0$, we construct a sequence $(v^n, \varepsilon^n), n \geq 0$ that is an approximation of the solution $(v(t,r),\ \varepsilon(t,r))$. At each time step, we have to solve a spatial problem, that we subdivide in two parts: 1) compute the velocity from the forces on the boundary and the momentum balance equation (solved with a finite element method); 2) move the boundary according to this velocity field and transport the deformation tensor.

Supplementary References

1 Bingham, E. C. *Fluidity and Plasticity* (McGraw-Hill, 1922).

2 Papanastasiou, T. C. Flows of Materials with Yield. *Journal of Rheology (1978-present)* **31**, 385-404, doi:doi:http://dx.doi.org/10.1122/1.549926 (1987).
